# Ineffectiveness of tactile gating shows cortical basis of nociceptive signaling in the Thermal Grill Illusion

**DOI:** 10.1038/s41598-018-24635-1

**Published:** 2018-04-26

**Authors:** E. R. Ferrè, G. D. Iannetti, J. A. van Dijk, P. Haggard

**Affiliations:** 1 Institute of Cognitive Neuroscience, University College London, London, UK; 2 Department of Psychology, Royal Holloway University of London, London, UK; 3 Department of Neuroscience, Physiology and Pharmacology, University College London, London, UK

## Abstract

Painful burning sensations can be elicited by a spatially-alternating pattern of warm and cold stimuli applied on the skin, the so called “Thermal Grill Illusion” (TGI). Here we investigated whether the TGI percept originates spinally or centrally. Since the inhibition of nociceptive input by concomitant non-nociceptive somatosensory input has a strong spinal component, we reasoned that, if the afferent input underlying the TGI originates at spinal level, then the TGI should be inhibited by a concomitant non-nociceptive somatosensory input. Conversely, if TGI is the result of supraspinal processing, then no effect of touch on TGI would be expected. We elicited TGI sensations in a purely thermal condition without tactile input, and found no evidence that tactile input affected the TGI. These results provide further evidence against a spinal mechanism generating the afferent input producing the TGI, and indicate that the peculiar burning sensation of the TGI results from supraspinal interactions between thermoceptive and nociceptive systems.

## Introduction

**Figure 1:**
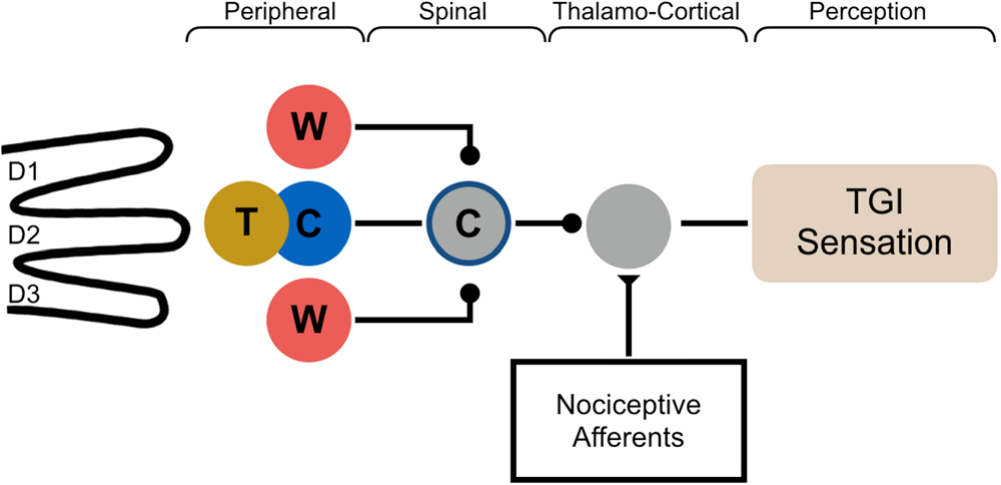
The classic model for TGI. The unmasking model of TGI (redrawn from *Craig and Bushnell*, 1994). Colours and letters indicate stimulus and skin temperatures, and the neural pathways corresponding to the temperature: “blue/C” = cold, “red/W” = warm, T = touch. In the classical model, there is tactile contact at all sites of thermal stimulation. In our experimental model, touch was delivered, or not delivered, on the middle finger alone.

The phylogenetically old nature of the nociceptive system is reflected in the large number of synaptic stations along its afferent pathways. This makes it particularly amenable to be modulated through interactions with other sensory systems, both spinally and supraspinally. A remarkable example is the paradoxical painful sensation of burning heat consequent to somatosensory stimulation with an alternating pattern of innocuous warm and cold bars, known as *Thermal Grill Illusion* (TGI)^1^. TGI has been traditionally explained by the unmasking of the activity of polymodal nociceptive lamina I spinothalamic neurons (Heat – Pinch – Cold, HPC, nociceptors) due to a reduction of the inhibition supplied by thermoceptive neurons that normally code for cold temperature (Fig. 1)^1^. The reduced activity in the cold pathway is caused, in turn, by spatial summation of neurons coding warmth^1^, which inhibit neurons coding cold. The consequent enhancement of afferent signaling by HPC neurons would produce the burning pain sensation. The sensation of burning pain that characterizes the TGI can be quantified as a subjective overestimation of the temperature of the cold stimuli within the grill^1^.

This theory emphasises modulation of ascending signals in a nociceptive HPC pathway originating in the spinal cord. However, later studies showed TGI-like interactions across dermatome boundaries^2^, and we have recently demonstrated that TGI is modulated by body posture (i.e. it disappears when the alternating pattern of warm and cold is disrupted by crossing the fingers)^3^. These observations provide strong evidence in favour of a cortical contribution to TGI. However, to our knowledge, few studies have specifically investigated whether the signal giving rise to TGI pain originates spinally or more centrally. Thus, the question whether the cold-nociceptive interaction occurs spinally or supraspinally, or both, remains unresolved. Here we address this question using the physiological concept of tactile-nociceptive *gate control*^4^. Gate control postulates a basic, powerful mechanism through which pain is generated and controlled. While the detailed implementation remains controversial^4^, the core finding, namely that touch can powerfully inhibit nociception at spinal levels, is widely accepted^5,6^, and recent animal studies have identified the specific cellular mechanisms that open and close the “gate”^7^. On the basis of this postulate we explored whether the nociceptive signaling in TGI arises at spinal or cortical levels by contrasting the intensity of the TGI percept when concurrent non-nociceptive somatosensory stimulation either is or is not present. If TGI originates at spinal levels, for example by activation of lamina I HPC nociceptive neurons^8^, then it seems likely that, like other nociceptive signals in the spinal cord, it should be reduced by concurrent non-nociceptive input. Conversely, finding no effect of tactile input on TGI would cast doubt on the involvement of spinal nociceptive signaling in TGI, and point instead to supraspinal interactions between thermoceptive and nociceptive systems.

The conventional TGI is based on mechanical contact between the skin and hot and cold stimulators. Tactile input is always present, so the effects of presence/absence of touch on TGI cannot be compared. We therefore developed a novel radiant thermal stimulation arrangement that allowed us to deliver either thermo-tactile or purely thermal TGI stimuli. We first replicated classical TGI sensations in the purely thermal condition, i.e. in absence of large-diameter afferent input (Experiment 1). Crucially, we observed that the presence or absence of simultaneous large-diameter cutaneous input had no reliable effect on a quantitative measure of TGI intensity (Experiment 2).

## Results

### Experiment 1: effectiveness of contactless TGI sensation

Nine healthy right-handed participants took part in Experiment 1. Thermal radiant stimuli were applied on the index, middle and ring fingers to produce either a neutral – cold – neutral (Baseline) or warm – cold - warm (TGI) pattern. The middle (target) finger was always stimulated with “radiant cold” induced by radiant heat transfer from the finger to some immediately adjacent dry ice^9^. The index and ring fingers were stimulated with neutral radiation in the Baseline condition and with warm radiation in the TGI condition. Single finger temperature matching confirmed that participants could clearly detect radiant cold and radiant warm stimuli when individually applied, and that these stimuli were not painful when tested individually (SI and Tables S1). Skin temperature recording showed the expected decreases for cold radiant stimuli and increases for warm radiant stimuli in skin temperature immediately after stimulation, confirming the effectiveness of thermal stimulation (SI and Tables S2). Our stimulation produced temperature changes comparable to previous TGI experiments^3^.

**Figure 2:**
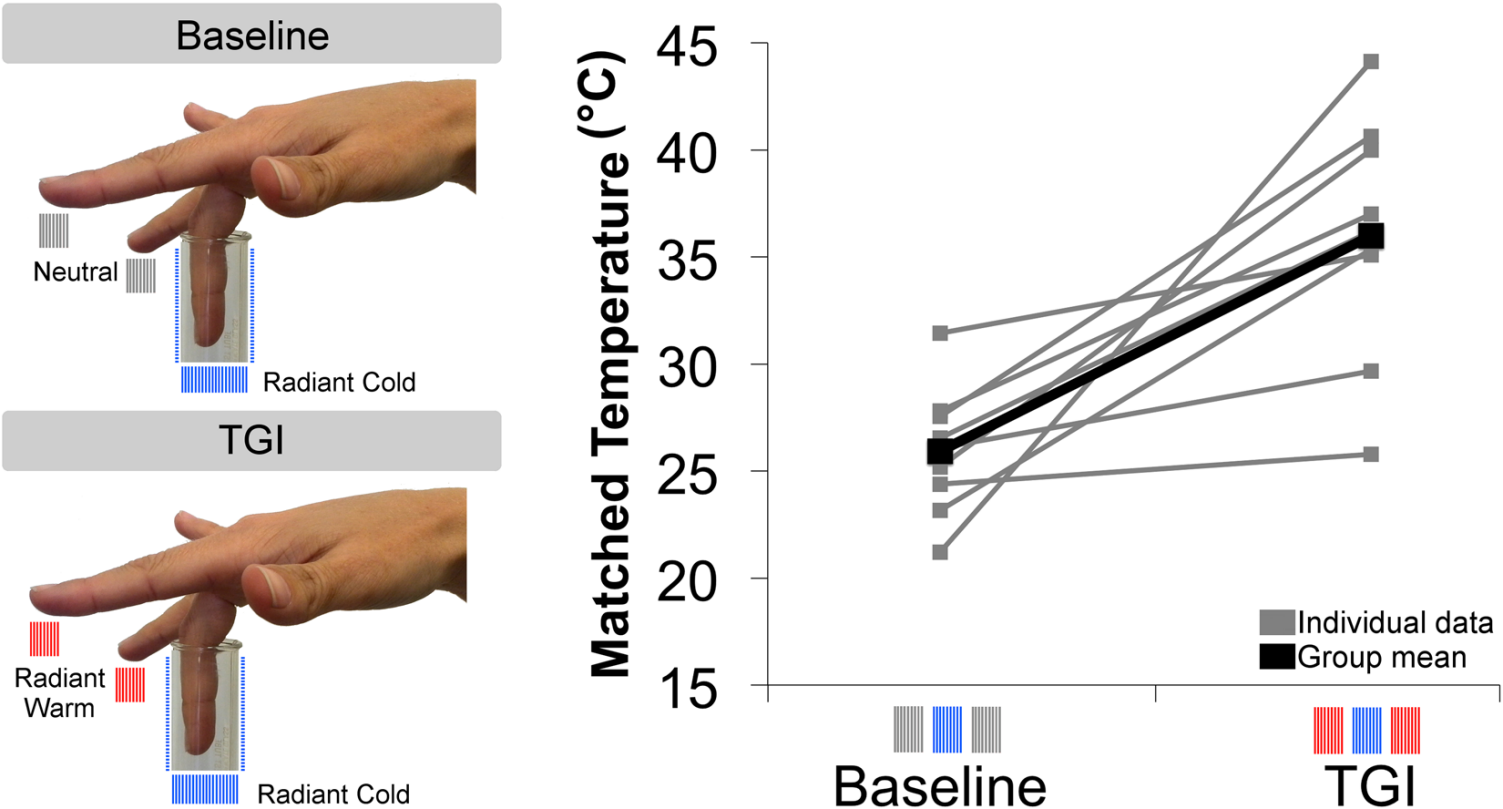
Contactless TGI evokes temperature overestimation. Methods and results for Experiment 1. Left panel. Thermal radiant stimuli were applied on the index, middle and ring fingers to produce either a neutral – cold – neutral (Baseline) or warm – cold - warm (TGI) pattern. Thermal radiant stimuli were delivered by dry ice and boiling water. Participants were instructed to insert the fingers in three different glass tubes. Each glass tubes were inserted in thermal isolating containers. The containers were filled with dry ice (approx. −50 °C), hot water (approx. 75 °C) or neutral temperature water (approx. 30 °C). The middle (target) finger was always stimulated with “radiant cold” induced by radiant heat transfer from the finger to the adjacent dry ice. The index and ring fingers were stimulated with neutral radiation in Baseline condition and with warm radiation in TGI condition. Right panel. Temperature was overestimated during TGI compared to Baseline as revealed by the temperature matching.

TGI was quantified with a temperature matching procedure^10^, in which the temperature of a thermode probe placed on the tip of the nose was gradually swept up or down, and the participant indicated when its temperature was felt to match that of the middle finger. Positive matching error indicated that the middle finger felt hotter than veridical (i.e., overestimation). A direct comparison between middle finger temperature matching in the Baseline and TGI condition revealed a significant difference between experimental conditions (t(8) = −4.494, p = 0.002; Cohen’s *d* = 2.248). Temperature was overestimated during TGI compared to Baseline (Fig. 2) in all participants. The consistent overestimation of cold temperature, comparable to that in previous TGI studies, suggests that purely thermal, contactless stimulation can effectively induce the burning hot sensation that characterises TGI.

### Experiment 2: tactile gating does not inhibit TGI

**Figure 3:**
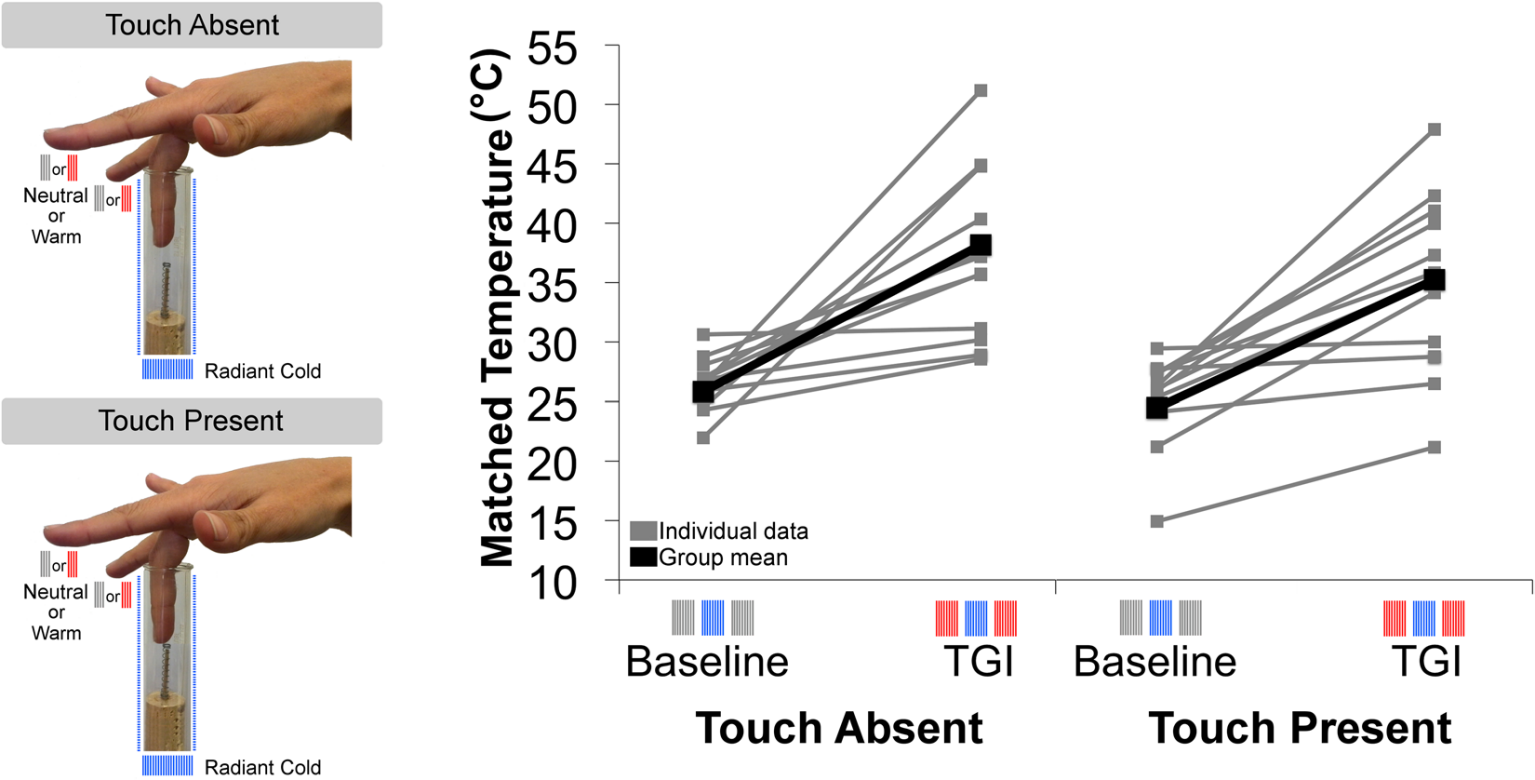
Tactile stimuli do not affect TGI sensation. Methods and results for Experiment 2. Left panel. Thermal radiant patterns of neutral-cold-neutral (Baseline) and warm-cold-warm (TGI) were delivered on participant’s right hand. In touch present trials a cork was inserted in the glass tube. A metallic spring was attached to it. Inside the spring a shorter wooden stick was also present. Participants were instructed to touch with the fingertip and exert a force to the spring until when they were able to detect the stick. Right panel. Temperature of the middle finger estimated by nose-matching confirmed that the overestimation for the middle finger in the touch absent condition did not differ significantly from the overestimation in the touch present condition.

Our core scientific question was whether TGI would be modulated by tactile input. We factorially combined tactile contact and TGI stimuli, in a 2 (tactile stimulus absent/present) × 2 (Baseline/TGI) design (Fig. 3). 12 new healthy right-handed participants took part in Experiment 2. The index, middle and ring fingers of the right hand were stimulated with thermal radiant or neutral stimuli, as before. In the touch present condition, the temperature matching procedure was performed while the middle finger of the right hand received innocuous mechanical tactile stimulation, from a spring-loaded stick touched lightly against the fingerpad. In the touch absent condition, no tactile stimulation was delivered.

Skin temperature data for each condition are shown in Table S3. Actual skin temperature decreased/increased immediately after TGI stimulation as expected, but was not influenced by the presence vs. absence of tactile stimulation in either Baseline (t(11) = −1.100, p = 0.295; Cohen’s *d* = 0.55) or TGI (t(11) = −0.883, p = 0.396; Cohen’s *d* = 0.44) (Table S3) conditions. A 2 × 2 repeated measures ANOVA on temperature of the middle finger estimated by nose-matching showed a main effect of TGI (F(1,11) = 27.659, p < 0.001 η^2^_P_ = 0.715), no effect of touch (F(1,11) = 1.399, p = 0.262 η^2^_P_ = 0.113) and no significant interaction between TGI and touch (F(1,11) = 0.350, p = 0.566 η^2^_P_ = 0.031) (Fig. 2). Interestingly, the overestimation for the middle finger in the touch absent condition did not differ significantly from the overestimation in the touch present condition (mean overestimation touch absent condition = 10.71 °C, SD = 8.10 °C; mean overestimation touch present condition = 9.74 °C, SD = 6.39 °C; t(11) = 0.590, p = 0.567; Cohen’s *d* = 0.28). In principle, this null result might just reflect low statistical power. However, tactile gating of nociception typically shows large effect sizes. For example, Mancini *et al*.^6^ reported that the probability of detecting a nociceptive stimulus was significantly reduced when touch was delivered concomitant with a pain stimulus, and the estimated effect size was Cohen’s *d* = 0.86. Similarly, the effect size for the reduced sensitivity induced by touch has also been reported to be very large, i.e. Cohen’s *d* = 1.95^5^. We thus applied a Bayesian repeated measures ANOVA (JASP, version 0.8.0.1; JASP Team 2016, University of Amsterdam to determine whether our data supported the null hypothesis^11^. The estimated model showed evidence against the interaction between TGI and Touch (BF10 = 0.368). That is, the data were almost three times more likely under the hypothesis of no tactile gating than under a hypothesis of tactile gating, approaching the ‘moderate’ level of evidence in categorical interpretations of Bayes Factors.

## Discussion

TGI was first described by Thunberg in 1896. He demonstrated that applying spatially adjacent cold (24 °C) and warm (44 °C) stimuli through interlaced coils produced a distinctive synthetic heat sensation^12^. He described this as the sensation that arises when the skin is exposed to a temperature at which just a minor increase will cause pain. TGI has proved valuable as an experimental model of pain, because it can be evoked by precisely controlled stimuli, does not involve tissue damage, and can be measured quantitatively as a bias in temperature perception for the cold stimuli within the grill arrangement. In recent years, it has become a primary research method for investigating the neural mechanisms underlying pain perception.

The classical experimental model for TGI entails a somatosensory input from non-nociceptive Aβ-afferents, elicited by the mechanical contact between the skin and hot and cold stimulators. We developed a novel radiant thermal stimulation arrangement that allowed us to deliver purely thermal TGI stimuli. Our stimuli are therefore different from the classic TGI arrangement with its multiple, interleaved bars. In particular, our stimuli were applied to the index, middle and ring fingers, rather than to a continuous skin region, and did not involve the high spatial frequency of alternating thermal stimuli of the classical TGI apparatus. Factors such as the total area stimulated, the spatial frequency of warm-cold-warm alternation, or some distinctive innervation of the specific skin regions we stimulated could potentially underlie the differences between our results and classical TGI studies. Nevertheless, our results with the radiant thermal stimulation arrangement recall previous TGI measures (Experiment 1). In particular, we demonstrated the characteristic ‘paradoxical heat’, or increase in perceived temperature of a skin region stimulated by cold, when surrounded by warm stimulation^13^. The magnitude of this effect was greater in the present study than in other previous studies in our lab using conventional contact stimulation via thermodes^3^.

Based on our results, we suggest that TGI is a purely cortical phenomenon, which may occur without any ascending nociceptive signal from the spinal cord. This proposal is supported by previous evidence that TGI sensation disappears when the alternating pattern of warm and cold is disrupted by modulating the body posture while keeping the somatotopical afferent pattern unchanged^3^. Thus, these results argue against the unmasking theory of Craig and Bushnell^1^, who proposed that burning pain elicited by C fibers occurs because a nociceptive pathway driven by HPC neurons is normally inhibited by thermoceptive Aδ fibers that produce the sensation of cool (COOL neurons) at spinal level^1^. In TGI, the normal inhibition of the nociceptive pathway by COOL is itself inhibited due to summation of signals in the warm pathway for surrounding skin regions. This leads to an unmasking of the nociceptive HPC pathway, and thus to the sensation of burning and pain.

In a series of papers, Craig and colleagues characterised the polymodal nociceptive HPC neurons in lamina 1 of the spinal cord^14–16^, and identified them as the key source of ascending spinothalamic signals that produce the “burning” pain that characterises TGI^1^. The TGI would then be the perceptual correlate of the HPC signal, unmasked by the loss of COOL inhibition at a thalamocortical level. Our observations raise the question of whether the ascending signals from HPC neurons would indeed be inhibited by tactile input, consistent with the general principle of tactile gating^4–6^. To our knowledge, no previous study has examined this question directly, although early studies found that A-fibre mechano-heat nociceptors in the dorsal horn of the spinal cord, which strongly responded to noxious thermal and mechanical stimuli including radiant heat, could indeed be inhibited by large-fibre input^17^. More recent physiological studies identified specific molecular pathways whereby tactile input closes the gate on spinal nociceptive neurons^18^. These lines of evidence predict that HPC signalling, like other nociceptive signals, should be strongly inhibited by touch, although HPC neurons, unlike other nociceptive-specific lamina 1 neurons, are relatively insensitive to mechanical force applied to the skin^19^. Our results are consistent with such a lack of tactile modulation of HPC: the failure to find any tactile gating of TGI suggests that HPC afferent signals may not be necessary for the effect. To the best of our knowledge, specific evidence regarding tactile gating of HPC signals is lacking, since all previous studies of identified HPC neurons appear to have used to use mechanical contact to deliver noxious thermal stimulation. However, the same mechanisms can be tested psychophysically in humans. If HPC neurons indeed underlie the TGI, and if these neurons do show the tactile gating that characterises other nociceptive afferents, then TGI should be *stronger* for radiant, non-contact thermal stimulation than for conventional thermal contact stimulation. Specifically, our radiant, non-contact TGI condition should doubly unmask ascending spinothalamic inputs thought to drive pain sensation. First, the absence of mechanical contact should cause loss of normal spinal gating of nociceptive afferents by touch. Second, the warm-cool-warm arrangement should cause loss of thalamocortical inhibition of HPC signalling by the COOL pathway. TGI sensations should thus be increased in a non-tactile, compared to a tactile arrangement. Our results did not support for this hypothesis. Given the ubiquity and strength of the tactile gating of pain^4–6^, absence of any tactile gating of TGI raises the question of whether a modulation of the afferent spinothalamic nociceptive signalling contributes to the TGI at all. Our results suggest that afferent signals evoked by TGI at earlier, spinal stages would be purely thermal, and not nociceptive. Tactile modulation of pain has traditionally been attributed to spinal mechanisms^4^, but cortical interactions may additionally be involved. The relative importance of spinal and supraspinal mechanisms remains controversial, but recent studies of radiant heat pain perception in humans confirm that an important spinal component exists^5,6^. However, our finding of no tactile effect suggests that the signals underlying the TGI are largely spared from the effects of tactile interactions at all levels.

We measured the TGI by temperature matching. Participants reported when a thermode applied to the nose felt to have the same temperature as the middle finger. The TGI therefore corresponds to an overestimation of temperature. Temperature measures of TGI have been used before, either alone^3,10^, and in conjunction with pain ratings^13^. They have the advantage of quantifying the TGI in physical units, which can then be compared across skin regions, across participants, and across studies, and avoid the notorious issues of defining what a *painful* sensation is and how it can be quantified. For this reason we did not ask participants to report explicitly whether they felt pain, also because the issue of whether the TGI elicits a genuine sensation of pain is hotly debated. The answer to this question must be very strongly dependent on what an individual participant defines as ‘pain’. This is inherently subjective, and highly variable.

Moreover, the spatial arrangement of the alternated innocuous warm and cold input used to elicit the TGI entailed the stimulation of smaller skin regions than other, classic TGI studies – which might well lead to less painful sensations. Our approach has been to avoid the ambiguous psychometric issues surrounding the definition of pain, in favour of a conceptually and scientifically simpler comparison between two thermal stimuli. The thermal sensations occurring in the TGI are recognised as strongly linked to pain experience, and to neurophysiology of the nociceptive system^1^. In conclusion, our results provide further evidence against a spinal mechanism generating the afferent input producing the TGI, and indicate that the burning sensation of the TGI is consequent to supraspinal interactions between thermoceptive and nociceptive systems.

## Methods

### Participants

Nine healthy right-handed participants (9 female, mean age ± SD: 24.22 ± 4.26 years) volunteered for Experiment 1 and other twelve healthy right-handed participants (12 female, mean age ± SD: 24.44 ± 2.07 years) for Experiment 2. Written informed consent was obtained from all participants. Exclusion criteria were sensitive skin on the hands (e.g., eczema), and analgesic medication (i.e., paracetamol, aspirin, ibuprofen, codeine) or recreational drug consumption in the last 24 hours. The experimental protocol was approved by the research ethics committee of University College London. The study adhered to the ethical standards of the Declaration of Helsinki.

### Radiant Thermal Stimulation

The index, middle and ring fingers of the right hand were stimulated with thermal radiant stimuli for 60 s. The thermal distribution was selected to make a warm – cold – warm pattern. The experimenter positioned the participant’s right hand on a support, and instructed participants to insert the fingers in three different glass tubes. Each glass tubes were inserted in thermal isolating containers. The containers were filled with dry ice (approx. −50 °C), boiling water (approx. 75 °C) or neutral temperature water (approx. 30 °C). The middle finger always received “radiant cold” stimuli, by placing a volume of dry ice close to the middle finger, and allowing the finger to cool by radiant heat exchange into the dry ice^9^. The other two fingers, received either radiant warm by boiling water (TGI condition) or neutral stimuli (Baseline condition) (Fig. 1). These stimuli induced transient decreases/increases in skin temperature higher than the thermal discrimination threshold (>1 °C;^20^), and readily discriminable, but lower than pain threshold^21,22^. Participants were instructed to do not touch the tubes. Washable colorant applied to the fingers control for it. After the thermal stimulation the experimenter removed each glass tubes from the containers and check for the presence of colorant on the tubes. Pictures were taken for each tube in each trial.

### Experiment 1: Contactless TGI

Verbal and written instructions were given to participants at the beginning of the experiment. Each participant completed two different tasks in a fixed order. First, participants performed a single finger thermal perception task, which served to confirm participants’ ability to correctly perceive cold and warm radiant stimuli. Then, participants performed the temperature-matching task.

#### Single finger thermal perception task

Participants were seated on a comfortable chair in a silent, temperature-controlled room (24 °C). They were asked to place their right hand over a custom build support, which hold the finger over the radiant thermal stimuli. One thermal radiant stimulus was delivered on the index, middle or ring finger of the right hand. Baseline and TGI temperatures were used. Three measures for each finger were collected. The finger stimulated was randomized within participant, while thermal condition (Baseline/TGI) order was counterbalanced between participants.

At the beginning of each trial, participants placed their right hand for 60 seconds over a thermal plate (Igloo 7200, Electron Dynamics Ltd) to set skin temperature at a constant baseline level. Skin temperature was measured by an infrared thermometer (Precision Gold N85FR), and found to conform to the intended baseline (range: 28 °C – 32 °C). Next, participant inserted the target finger (index, middle or ring) in the corresponding glass tube. Thermal stimulation of the fingers was delivered continuously for 60 s. After the thermal stimulation period, a single finger matching-temperature procedure started. A 13 mm circular diameter Peltier-type thermode (NTE-2A, Physitemp Instruments Inc) was mounted on a stand touching the tip of the nose. The temperature of this probe was initially set at 30 °C. Participant was asked to compare the temperature perceived on the nose with that perceived on the fingers of the right hand. If participant perceived different temperatures, the experimenter either decreased or increased the temperature of the left finger probe, according with the direction of the perceived mismatch. The matching temperature was increased or decreased until the participant felt that the two temperatures were similar. Skin temperature was recorded immediately after stimulation (post-stimulation). Participants’ hands were placed on a table, and the temperature recorded by the experimenter. For the entire duration of the task participants were blindfolded.

#### Temperature-matching task

TGI produces unusual sensations, with a characteristic quality of heat, often described as “burning pain”^23^. We quantified TGI using temperature matching^8^, rather than qualitative judgements. The temperature of a thermode probe on the tip of the nose was gradually swept up or down, and the participant indicated when its temperature was felt to match that of the middle finger, by pressing a key with their left hand. Positive matching errors indicated that the middle finger felt hotter than veridical (i.e., overestimation). We chose perceived temperature as a dependent variable, because it gives quantitative data, does not involve any subjective interpretation of what counts as ‘pain’, is commonly reported in nociceptive sensations using thermal grill stimulation^24^, has been reliably used before in matching tasks^9^, and reflects the same continuous, underlying mechanism as pain judgement^22^.

Thermal radiant patterns of neutral – cold – neutral (Baseline) and warm – cold – warm (TGI) were delivered on participant’s right hand. Order of thermal condition (Baseline/TGI) was counterbalanced between participants. Participants performed the temperature-matching procedure three times for both Baseline and TGI conditions. Skin temperature was recorded immediately before (pre-stimulation) and immediately after (post-stimulation) radiant stimulation. Order of experimental conditions was counterbalanced across participants.

### Experiment 2: Contactless TGI

Verbal and written instructions were given to participants at the beginning of the experiment. Participants were seated on a comfortable chair in a silent, temperature-controlled room (24 °C). The design combined tactile stimulation (present/absent) and thermal condition (Baseline/TGI). In touch present trials a cork was inserted in the middle finger glass tube. A metallic spring was attached to it. Inside the spring a shorter wooden stick was also present. Participants were instructed to touch with the fingertip and exert a small downward pressure on the spring until they were able to clearly feel the stick touching their skin (tactile contact force exerted was about 2 N). Participants hold their finger on the wooden stick so that there was sustained pressure, which induces a continuous mechanical stimulation, possibly affecting SA1 tactile input. Experimental procedure was as in Experiment 1. Order of condition (Baseline/TGI) was counterbalanced between participants. Three measures for each thermal condition were collected.

## Electronic supplementary material

Supplementary Material
