# Supplementary material for: Ineffectiveness of tactile gating shows cortical basis of nociceptive signaling in the Thermal Grill Illusion
Source: Sci Rep. 2018 Apr 26;8:6584. doi: 10.1038/s41598-018-24635-1 (PMC5919908; doi:10.1038/s41598-018-24635-1)
Supplement: Supplementary file 1 — Supplementary Material [file 41598_2018_24635_MOESM1_ESM.docx]

Ineffectiveness of tactile gating shows cortical basis of nociceptive signaling in the Thermal Grill Illusion

E.R. Ferrè, G.D. Iannetti, J. Van Dijk, P. Haggard

**Supplementary Information**

**Effectiveness of thermal radiant stimulation**

First, we checked whether the radiant thermal stimuli produced a measurable increase in skin temperature. We entered post-stimulation skin temperature values in a series of direct comparisons between fingers. In the Baseline, clear differences in skin temperature were found for index (neutral) and middle (cold) fingers (t(8)=8.420; p<0.001) and for ring (neutral) and middle (cold) fingers (t(8)=8.608; p<0.001), no differences were found between index (neutral) and ring (neutral) fingers (t(8)=-0.381; p=0.713). In TGI, differences were found for index (warm) and middle (cold) fingers (t(8)=8.943; p<0.001) and for ring (warm) and middle (cold) fingers (t(8)=8.956; p<0.001), while no differences between index (warm) and ring (warm) fingers (t(8)=1.655; p=0.137) were observed. Skin temperature comparison between Baseline and TGI showed significant differences for the index and ring fingers (t(8)=-2.900; p=0.020 and t(8)=-3.004; p=0.017 respectively). No differences emerged for the middle finger (t(8)=-0.142; p=0.891). Thus, our stimulation intensities produced an increase/decrease in the skin temperature of each finger, as expected.

Next, we checked whether participants correctly *perceived* the different thermal levels. A series of comparisons was performed on the temperature matching estimates. In Baseline, cold stimuli on the middle finger were perceived colder than neutral stimuli on the index (t(8)=2.567; p=0.033) and ring finger (t(8)=4.111; p=0.003). No differences emerged between index (neutral) and ring (neutral) fingers (t(8)=0.887; p=0.401). In TGI, cold stimuli on the middle finger were perceived colder compared warm stimuli on the index (t(8)=8.795; p<0.001) and for ring fingers (t(8)=8.517; p<0.001). No differences were found between index (warm) and ring (warm) fingers (t(8)=0.902; p=0.393). Participants correctly perceived the index and ring fingers as warmer in TGI condition compared to Baseline (t(8)=-3.922; p=0.004 and t(8)=-3.431; p=0.009 respectively). No difference was found for the middle finger, which received the same cold radiant stimulation in both experimental conditions (t(8)=1.778; p=0.972). Thus, the stimulation induced proper variations in temperature perception on each finger.

**Skin temperature during the temperature-matching task (Experiment 1)**

We computed the difference between post-stimulation and pre-stimulation skin temperature, and we entered those values in a series of direct comparisons. In the Baseline, differences in skin temperature were found for index (neutral) and middle (cold) fingers (t(8)=9.032; p<0.001) and for ring (neutral) and middle (cold) fingers (t(8)=9.667; p<0.001), no differences were found between index (neutral) and ring (neutral) fingers (t(8)=-0.460; p=0.658). In TGI, differences were found for index (warm) and middle (cold) fingers (t(8)=9.314; p<0.001) and for ring (warm) and middle (cold) fingers (t(8)=8.358; p<0.001), while no differences between index (warm) and ring (warm) fingers (t(8)=2.018; p=0.078) were observed. Skin temperature comparison between Baseline and TGI showed significant differences for the index and ring fingers (t(8)=-8.280; p<0.001 and t(8)=-6.314; p<0.001 respectively). No differences were found for the middle finger (t(8)=0.211; p=0.838).

**Table S1.**

**Experiment 1: single finger matching**

Mean and SD of actual and judged skin temperature for each finger in each experimental condition.

|  |  | **Finger** |  |  |
| --- | --- | --- | --- | --- |
|  |  | Index | Middle | Ring |
| **Baseline** |  |  |  |  |
| Actual | Mean | 26.62 | 22.11 | 26.76 |
|  | SD | 5.53 | 5.50 | 5.31 |
| Judged | Mean | 30.73 | 26.29 | 29.58 |
|  | SD | 3.24 | 3.33 | 3.09 |
| **TGI** |  |  |  |  |
| Actual | Mean | 32.49 | 22.33 | 31.72 |
|  | SD | 2.98 | 4.50 | 3.21 |
| Judged | Mean | 36.09 | 26.32 | 35.22 |
|  | SD | 2.43 | 1.67 | 2.98 |

**Table S2.**

**Experiment 1: skin temperature.**

Mean and SD of skin temperature for each finger immediately before and after stimulation

|  |  | **Finger** |  |  |
| --- | --- | --- | --- | --- |
|  |  | Index | Middle | Ring |
| **Baseline** |  |  |  |  |
| Pre-Stimulation | Mean | 29.20 | 29.09 | 29.24 |
|  | SD | 3.20 | 3.33 | 3.41 |
| Post-Stimulation | Mean | 28.17 | 23.31 | 28.28 |
|  | SD | 3.80 | 4.91 | 4.02 |
| **TGI** |  |  |  |  |
| Pre-Stimulation | Mean | 29.24 | 28.71 | 28.56 |
|  | SD | 3.41 | 3.16 | 3.65 |
| Post-Stimulation | Mean | 32.25 | 22.61 | 31.96 |
|  | SD | 2.54 | 5.52 | 2.55 |

**Table S3.**

**Experiment 2: skin temperature.**

Mean and SD of skin temperature for each finger immediately before and after stimulation

|  | |  |  | **Finger** |  |  |
| --- | --- | --- | --- | --- | --- | --- |
|  | |  |  | Index | Middle | Ring |
| **Touch Absent** | | | | | | |
|  | **Baseline** | |  |  |  |  |
|  | Pre-Stimulation | | Mean | 31.26 | 31.43 | 32.10 |
|  |  | | SD | 1.10 | 1.69 | 1.56 |
|  | Post-Stimulation | | Mean | 30.95 | 25.43 | 31.23 |
|  |  | | SD | 1.12 | 3.26 | 1.21 |
|  | **TGI** | |  |  |  |  |
|  | Pre-Stimulation | | Mean | 31.83 | 32.23 | 32.90 |
|  |  | | SD | 1.00 | 1.30 | 1.05 |
|  | Post-Stimulation | | Mean | 34.10 | 26.46 | 34.66 |
|  |  | | SD | 1.02 | 2.29 | 0.74 |
| **Touch Present** | | | | | | |
|  | **Baseline** | |  |  |  |  |
|  | Pre-Stimulation | | Mean | 31.80 | 32.09 | 32.55 |
|  |  | | SD | 1.01 | 1.47 | 1.23 |
|  | Post-Stimulation | | Mean | 31.46 | 27.26 | 31.87 |
|  |  | | SD | 1.00 | 2.68 | 1.27 |
|  | **TGI** | |  |  |  |  |
|  | Pre-Stimulation | | Mean | 31.86 | 31.99 | 32.77 |
|  |  | | SD | 0.90 | 1.35 | 1.14 |
|  | Post-Stimulation | | Mean | 34.36 | 26.17 | 34.67 |
|  |  | | SD | 0.98 | 2.06 | 0.74 |
